# Trendelenburg maneuver to predict fluid responsiveness in patients under mechanical ventilation with spontaneous breathing: a prospective study

**DOI:** 10.1186/s12871-026-03706-1

**Published:** 2026-02-21

**Authors:** Nattachai Hemtanon, Nattaya Raykateeraroj, Suneerat Kongsayreepong, Thassayu Yuyen, Pasith Boorapapon, Yukiko Hikasa, Suchanun Lao-amornphunkul, Nuanprae Kitisin

**Affiliations:** 1 Department of Anesthesiology, Faculty of Medicine Siriraj Hospital, Mahidol University, 2 Wanglang Road Bangkoknoi, Bangkok, 10700 Thailand; 2 Department of Intensive Care, Austin Hospital, Heidelberg, VIC Australia

**Keywords:** Trendelenburg position, Cardiac output, Spontaneous breathing, Hemodynamic monitoring, Volume therapy, Intensive Care Units, Predictive value of tests, Sensitivity and specificity

## Abstract

**Background:**

Both hypovolemia and fluid overload are associated with adverse outcomes in critically ill patients, yet many methods for assessing fluid responsiveness have limitations. The Trendelenburg maneuver, which transiently increases cardiac preload through a head-down tilt and operates on the same principle as passive leg raising but is easier to perform and more comfortable, may offer a practical adjunct in light-sedated patients under mechanical ventilation with spontaneous breathing activity, a common clinical scenario.

**Methods:**

In this single-center, prospective study, mechanically ventilated adults with spontaneous breathing and signs of tissue hypoperfusion in a surgical intensive care unit (ICU) underwent a standardized sequence of hemodynamic assessments: supine baseline (T0), reverse Trendelenburg + 10° (T1), Trendelenburg − 13° (T2), return to supine before fluid loading (F0), and reassessment after a 4 mL/kg infusion of 5% albumin over 15 min (FL). Hemodynamics (cardiac index [CI], stroke volume variation [SVV], pulse pressure variation [PPV], central venous pressure [CVP]) were measured with FloTrac™/HemoSphere™. Fluid responsiveness was defined as a ≥ 10% increase in CI from F0 to FL. The diagnostic performance of Trendelenburg-induced hemodynamic changes (Δ values, T2–T1) and baseline variables was evaluated using the area under the receiver operating characteristic curve (AUROC), and gray-zone analysis was performed to quantify diagnostic uncertainty.

**Results:**

Thirty-eight patients were included; 21 (55%) were classified as fluid responders. A ΔCI > 0.15 L/min/m^2^ yielded an AUROC of 0.78 (95% confidence interval, 0.64–0.93), with 52.4% sensitivity and 82.4% specificity; however, more than 80% of patients fell within the gray zone. A Δ%CI > 6% showed similar performance, while ΔSVV and ΔPPV demonstrated lower but moderate accuracy. In sensitivity analyses, diagnostic performance decreased when fluid responsiveness was redefined as a ≥ 15% increase in CI, and was slightly lower among patients receiving norepinephrine < 0.1 µg/kg/min.

**Conclusions:**

The Trendelenburg maneuver was feasible and safe but showed limited and inconsistent diagnostic performance for predicting fluid responsiveness. With more than 80% of patients falling within wide gray zones, the test is inconclusive for most bedside decisions and should not be used as a stand-alone guide.

**Trial registration:**

Thai Clinical Trials Registry (TCTR20230704005) registered 4 July 2023.

**Supplementary Information:**

The online version contains supplementary material available at 10.1186/s12871-026-03706-1.

## Background

Optimal fluid management is a cornerstone of critical care, as both fluid overload and hypovolemia contribute to adverse outcomes in intensive care unit (ICU) patients [1–3]. Accurate prediction of fluid responsiveness is essential to guide individualized fluid therapy. Dynamic indices derived from heart–lung interactions, such as pulse pressure variation (PPV) and stroke volume variation (SVV), provide reasonable diagnostic accuracy. Their applicability, however, is restricted to patients under controlled mechanical ventilation—without spontaneous breathing or arrhythmias [4, 5]—a scenario encountered in only a minority of ICU patients.

Passive leg raising (PLR) has become a widely accepted method for assessing fluid responsiveness because it reliably produces a reversible autotransfusion, even in patients with spontaneous respiration [6, 7], with diagnostic performance confirmed in meta-analyses showing pooled sensitivity and specificity above 80% [8]. However, in the surgical ICU, its feasibility may be limited by postoperative pain, surgical wounds or drains, abdominal or spinal surgical contraindications, and incompatibility with certain types of beds and monitors [9].

The Trendelenburg maneuver (TM) —a head-down tilt that transiently increases preload — offers a more easily implemented alternative. Although TM and PLR operate on similar physiological principles, the preload shift generated by TM is more susceptible to interference from spontaneous breathing. The cephalad displacement of abdominal contents during TM can increase diaphragmatic load and amplify negative pleural-pressure swings, potentially masking or blunting the hemodynamic response.

Prior studies have shown promising accuracy, but all were conducted in deeply sedated, fully controlled-ventilation patients [10], including those with acute respiratory distress syndrome (ARDS) in prone position or receiving veno-arterial extracorporeal membrane oxygenation (VA-ECMO) [11, 12]. These settings eliminate spontaneous respiratory effort, making preload transmission during TM more predictable than in a lightly sedated patient who is partially breathing on their own.

Because the majority of postoperative surgical ICU patients are lightly sedated (often Richmond Agitation Sedation Scale; RASS 0 to − 1) and exhibit some degree of spontaneous breathing, the true diagnostic performance of TM in this pragmatic population remains unknown. Variable pleural pressures, irregular respiratory patterns, and diaphragm–abdominal interactions may reduce the accuracy of preload-induced maneuvers, but this has never been formally tested.

This study aimed to evaluate the diagnostic accuracy of cardiac index (CI) changes during the Trendelenburg maneuver, compared with fluid administration, for predicting fluid responsiveness among lightly sedated surgical ICU patients under mechanical ventilation with spontaneous breathing activity. We hypothesized that TM-induced hemodynamic changes would predict fluid responsiveness in this population.

## Materials and methods

### Study design and ethical approval

This single-center, prospective study was conducted in a 14-bed surgical ICU of a tertiary care hospital between July 2023 and May 2025. The ICU comprises two units, each with seven single-patient rooms, and primarily admits postoperative general surgical patients. Cardiothoracic, neurosurgical, and major trauma cases are managed in separate specialty ICUs; accordingly, the study population mainly represents a general postoperative surgical ICU cohort. The study was approved by the Siriraj Institutional Review Board (approval number Si 386/2023) and registered with the Thai Clinical Trial Registry (TCTR20230704005). Written informed consent was obtained from each patient’s legally authorized representative or next of kin at enrollment, when the patient was unable to provide consent due to critical illness. When patients subsequently regained capacity, written re-consent was obtained directly from them. The research was conducted in accordance with the Declaration of Helsinki and adhered to the Standards for Reporting of Diagnostic Accuracy Studies (STARD 2015) guidelines [13], as detailed in Supplementary Appendix I.

### Study population

After ICU admission, eligible participants were assessed. Patients were screened at least twice daily by the research team. Signs of inadequate tissue perfusion were evaluated at ICU admission using standardized bedside criteria. Capillary refill time was measured by applying pressure to the fingertip with a glass slide for 10 s. When a patient fulfilled all inclusion criteria, written informed consent was obtained and enrollment proceeded immediately. If signs of inadequate tissue perfusion developed later during the ICU stay, the patient was enrolled at the time the criteria were met.

Inclusion criteria were age ≥ 18 years; receiving mechanical ventilation with spontaneous breathing activity, confirmed by the presence of patient-triggered breaths during assisted-control ventilation or the patient's active participation while on pressure support ventilation; ability to cooperate with the maneuver, either awake or under sedation with RASS from −2 to + 1; and the presence of at least one sign of inadequate tissue perfusion, including mean arterial pressure (MAP) < 65 mmHg, heart rate (HR) > 100/minute, urine output < 0.5 mL/kg/h, skin mottling, capillary refill time > 2 s, or serum lactate > 2 mmol/L.

Exclusion criteria were refusal of participation by the patient or the patient’s representative; contraindications to the Trendelenburg maneuver, including elevated intracranial or intraocular pressure; contraindications to peripheral arterial cannulation; cardiogenic pulmonary edema; left ventricular ejection fraction (LVEF) < 30%; severe aortic or mitral valve dysfunction; persistent arrhythmias; pulmonary artery hypertension, defined as mean pulmonary arterial pressure > 25 mmHg; intra-abdominal hypertension; continuous muscle relaxant infusion; thrombosis of the inferior vena cava or major lower extremity veins; above-ankle amputation; allergy to or refusal of human albumin; and pregnancy.

### Study protocol and measurements

This was a non-blinded study, as investigators were aware of the intervention sequence; however, all outcomes were based on objective hemodynamic measurements obtained directly from the monitoring system. HR, respiratory rate, MAP from invasive arterial pressure, central venous pressure (CVP), oxygen saturation (SpO₂), and end-tidal CO₂ were continuously monitored using bedside multiparameter ICU monitors (Philips IntelliVue series, Philips Healthcare, Best, The Netherlands).

All patients had a radial arterial catheter connected to a FloTrac™ sensor (model MHD6, fourth-generation algorithm) interfaced with the HemoSphere™ advanced monitoring platform (model HEM1; Edwards Lifesciences, Irvine, CA, USA) for continuous uncalibrated pulse-contour analysis. To ensure measurement accuracy, the arterial pressure and CVP (if present) transducers were zeroed and secured at the phlebostatic axis and remained fixed at the patient’s bed throughout the reverse Trendelenburg and Trendelenburg maneuvers, with waveform quality visually inspected before each recording.

**Fig. 1:**
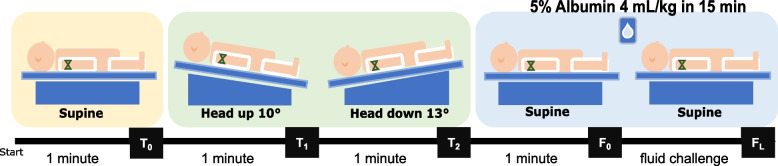
Study protocol timeline and intervention steps. Patients began in the supine position for baseline measurements (T0). Reverse Trendelenburg (T1) consisted of tilting the bed to + 10° head-up for 1 min, followed by − 13° Trendelenburg (T2) for 1 min. Patients were then returned to supine for 1 min (F0), followed by a fluid challenge with 4 mL/kg of 5% human albumin over 15 min (FL). Hemodynamic parameters were measured at each stage. T0, T1, T2, F0, and FL represent baseline, reverse Trendelenburg, Trendelenburg, pre-infusion, and post–fluid-loading time points, respectively

Preparatory measures were undertaken to minimize artefacts and ensure patient safety. This included confirmation of appropriate endotracheal cuff pressure and nasogastric tube suctioning, performed several minutes prior to the start of the maneuver. All patients were receiving invasive mechanical ventilation at the time of inclusion. Ventilator mode (pressure-controlled ventilation, volume-controlled ventilation, or pressure-support ventilation) was selected at the discretion of the treating intensivist and was kept unchanged throughout the protocol. The overall study protocol is illustrated in Fig. 1.

The protocol began once all monitoring devices had been verified to be connected, calibrated, and functioning properly. To minimize anxiety and agitation, the patient was informed of the steps before initiating the maneuver. The patient was then placed in the supine position, and baseline measurements (T0) were recorded after 1 min (ventilator mode, tidal volume (TV), respiratory rate, minute ventilation (MV), SpO₂, end-tidal CO_2_, and RASS). The bed was then tilted to a reverse Trendelenburg (10° head-up) position for 1 min (T1), followed by a Trendelenburg (13° head-down) position for 1 min (T2). A 1-min duration was selected because preload-modifying maneuvers reach their maximal hemodynamic effect within approximately 30–90 s and extending the observation period risks attenuation of the response due to baroreflex compensation [14]. Hemodynamic parameters were recorded at 20-s intervals. The maximum change from previous step was recorded for each hemodynamic variable—particularly the CI—during this phase, even if it occurred before the full minute elapsed; for example, if CI increased from 2.4 L/min/m^2^ at T1 to 2.4, 2.5, 2.7 L/min/m^2^ during T2, 2.7 L/min/m^2^ was recorded. Bed angles were limited to + 10° and − 13° by the maximum tilt capacity of our ICU beds.

The patient was then returned to the supine position for one minute (F0), during which pre-infusion hemodynamic measurements were taken. A fluid bolus of 5% human albumin (4 mL/kg) was subsequently administered over 15 min in the supine position, and hemodynamic variables were measured immediately at the end of the 15-min infusion (FL), without an additional post-infusion observation period. Five percent human albumin was chosen due to its rapid intravascular expansion effect and common use in fluid responsiveness studies, with colloids employed in 62% of fluid-challenge studies in critically ill patients [15, 16]. At each protocol time point (T0, T1, T2, F0, FL) a standardized hemodynamic dataset was recorded, including cardiac output (CO), CI, SVV, and PPV from the FloTrac™/HemoSphere™ system; CVP, HR, and MAP from the ICU monitor; and the level of sedation assessed using the RASS.

Fluid responsiveness was defined as an increase in CI of at least 10% from F0 to FL, measured using the FloTrac™/HemoSphere™ system. This cutoff was prespecified based on previous literature [17–20]. Although those studies primarily employed calibrated techniques (e.g., transpulmonary thermodilution) rather than uncalibrated pulse-contour analysis, one study by Messina et al. [21] assessed a 10% cutoff using an uncalibrated device (MostCare®, Vyetech Health, Padua, Italy). As the least significant change (LSC) of CI measurements with the fourth-generation FloTrac™/HemoSphere™ platform has not been formally established, we additionally performed a sensitivity analysis using a 15% threshold to align more closely with reported device precision.

Throughout the study period, no changes were permitted in fluid management, vasopressor use, sedation, or ventilator settings. The protocol included predefined termination criteria to ensure patient safety: severe hypotension or cardiovascular collapse requiring resuscitation, new-onset persistent arrhythmias, oxygen desaturation (SpO₂ < 90%), severe agitation (RASS ≥ + 2), or regurgitation.

### Data collection

Baseline demographic and clinical information included age, sex, body mass index (BMI), American Society of Anesthesiologists (ASA) physical status, Sequential Organ Failure Assessment (SOFA) score, Acute Physiology and Chronic Health Evaluation II (APACHE II) score, primary diagnosis, indication for ICU admission, and causes of inadequate tissue perfusion. Data on vasopressors and sedative use were also collected, including the type and dose administered. Baseline laboratory values included serum lactate and bicarbonate (HCO₃⁻) levels.

Respiratory parameters at enrollment included arterial blood gas values (pH, arterial oxygen partial pressure to inspired oxygen fraction ratio [PaO₂/FiO₂], and partial pressure of carbon dioxide [PaCO₂]) which were measured using an ABL800 FLEX blood gas analyzer; ventilator settings (positive end-expiratory pressure [PEEP], TV per predicted body weight [TV/PBW], and MV); and airway pressures, specifically peak inspiratory pressure (PIP).

Intra-abdominal pressure (IAP) was recorded at enrollment because intra-abdominal hypertension was an exclusion criterion. IAP was measured via the bladder using the standardized intravesical technique (50 mL normal saline instillation through the indwelling urinary catheter, measurement at end-expiration with the transducer zeroed at the mid-axillary line).

Adverse events observed during TM period were systematically recorded, including new-onset or sustained cardiac arrhythmia, oxygen desaturation (SpO_2_ < 90%), regurgitation, and agitation (RASS > + 2).

### Outcomes

The primary outcome was the ability of the Trendelenburg maneuver to predict fluid responsiveness in mechanically ventilated patients with spontaneous breathing activity. This was evaluated by comparing the maximal change in CI during TM—measured with the FloTrac™/HemoSphere™ system—with the CI response following fluid administration.

Secondary outcomes included the diagnostic performance of other hemodynamic variables during TM—such as SVV, PPV, and CVP—as alternative predictors of fluid responsiveness. Additionally, the predictive value of baseline parameters, as well as combinations of baseline and dynamic variables, was explored to enhance accuracy.

### Statistical analysis

A priori sample size estimation was performed using Hanley and McNeil’s method for receiver operating characteristic (ROC) curve analysis, assuming a null area under the ROC curve (AUROC) of 0.5, an expected AUROC of 0.75, a two-sided alpha of 0.05, and 90% power. This indicated that at least 38 patients (≈19 responders and 19 non-responders) were required. Statistical analyses were conducted in R (version 4.4.3, R Foundation for Statistical Computing, Vienna, Austria). Continuous variables were assessed for distribution by histogram and Q–Q plot. Normally distributed variables were reported as mean (standard deviation: SD) and compared using the independent t-test; non-normally distributed variables were reported as median (interquartile range: IQR) and compared using the Mann–Whitney U test. Categorical variables were summarized as counts (%) and compared using the chi-square or Fisher’s exact test, as appropriate. A *p*-value < 0.05 was considered statistically significant.

Longitudinal hemodynamic data (CI, MAP, SVV, PPV, CVP) were analyzed using linear mixed-effects models with fixed effects for group (responder vs non-responder), time (T0, T1, T2, F0, FL), and their interaction, and a random intercept for patient. CVP was available only in the subset of patients with a central venous catheter (*n =* 27); analyses involving CVP were performed using complete-case analysis without imputation and were considered exploratory, as CVP was not a primary predictor. Model-estimated marginal means with 95% confidence intervals were obtained. The group × time interaction provided the global test for between-group differences over time. Post-hoc pairwise comparisons were performed to identify significant differences between groups at specific time points. Model performance was compared using Akaike information criterion (AIC) and Bayesian information criterion (BIC). Model assumptions were assessed using Q–Q plots for residual normality, residual-versus-fitted value plots for homoscedasticity, and influence diagnostics.

ROC curve analyses were then performed (pROC package, R) [22] for absolute ΔCI, Δ%CI, ΔSVV, and ΔPPV during the Trendelenburg maneuver. The AUROC with 95% CI was calculated, and diagnostic accuracy metrics—including sensitivity, specificity, positive predictive value (PPV [diagnostic]), and negative predictive value (NPV)—were derived using MedCalc Statistical Software (version 22.009; MedCalc Software Ltd., Ostend, Belgium). Thresholds for the primary analysis and interpretation were chosen for bedside interpretability, with emphasis on high sensitivity or specificity rather than strict optimization by the Youden index [23] and were informed by previous studies [10, 11, 24] reporting comparable cutoff ranges of approximately 4–13% change for Trendelenburg-induced CI variations. For each parameter, the cutoff that maximized the Youden index (sensitivity + specificity − 1) was also calculated; these Youden-based thresholds are presented alongside the a priori, clinically oriented cutoffs used in the main analysis. Gray-zone analysis was conducted by identifying the interval in which both sensitivity and specificity were < 90%, and the proportion of patients within this interval was reported [25].

Two sensitivity analyses were prespecified. First, analyses were repeated in patients receiving norepinephrine at < 0.1 µg/kg/min. This cutoff was chosen because Monnet et al. reported that the FloTrac™ system was unreliable during norepinephrine titration at a mean dose of 0.16 µg/kg/min (IQR 0.04–0.41) [26]. By restricting to < 0.1 µg/kg/min, we aimed to evaluate device performance under conditions of minimal vasopressor influence. Second, fluid responsiveness was redefined as a ≥ 15% increase in CI, reflecting reported estimates of device precision [27].

## Results

### Population

**Fig. 2:**
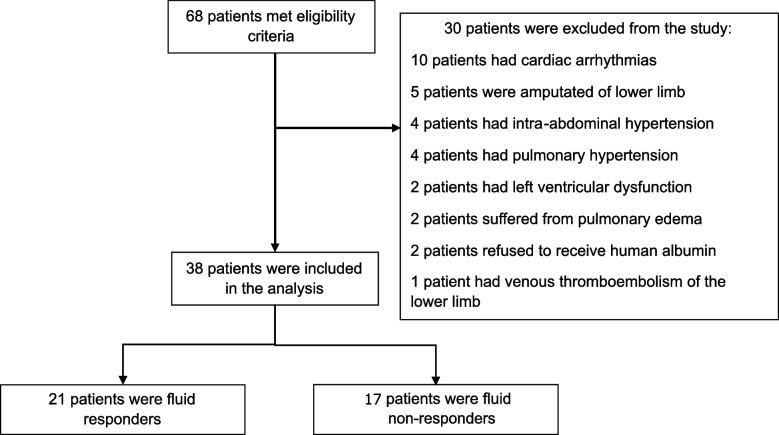
Study enrollment and analysis. The figure shows patient flow from screening to analysis. Sixty-eight patients were screened; 30 were excluded, leaving 38 patients included in the analysis (21 responders, 17 non-responders)

During the study period, 68 patients met the eligibility criteria. Of these, 30 patients were excluded, most commonly due to cardiac arrhythmias, leaving 38 patients in the final analysis (Fig. 2). Among them, 21 patients (55%) were classified as fluid responders, and 17 patients (45%) were fluid non-responders.

### Patient characteristics

The baseline characteristics are summarized in Table 1. Fluid responders and non-responders were generally comparable in terms of demographics (age, sex distribution, BMI, ASA physical status), surgical type, and ICU admission source. The distribution of shock etiology and indications for fluid evaluation was also similar between groups.

**Table 1:** Baseline characteristics of fluid responders and non-responders

Characteristics	Overall (*n =* 38)	Responder group (*n =* 21)	Non-responder group (*n =* 17)	p-value
Age (years)	66.8 ± 12.9	69.3 ± 14.2	63.6 ± 10.9	0.17
Female, n (%)	24 (63.2)	12 (57.1)	12 (70.6)	0.39
BMI (kg/m^2^)	23.8 ± 4.1	22.5 ± 2.5	25.3 ± 5.2	0.06
ASA physical status, n (%)				0.28
-ASA I	1 (2.6)	0 (0)	1 (5.9)	
-ASA II	7 (18.4)	2 (9.5)	5 (29.4)	
-ASA III	20 (52.6)	11 (52.4)	9 (52.9)	
-ASA IIIE	3 (7.9)	3 (14.3)	0 (0)	
-ASA IV	3 (7.9)	2 (9.5)	1 (5.9)	
-ASA IVE	4 (10.5)	3 (14.3)	1 (5.9)	
SOFA score	4 (2, 6.8)	3 (2, 5)	6 (3, 8)	0.01
APACHE II score	11 (9, 14.8)	9 (8, 16)	12 (10, 14)	0.29
Lactate (mmol/L)	3.22 ± 1.39	3.36 ± 1.24	3.07 ± 1.58	0.55
HCO₃⁻ (mmol/L)	18.9 ± 3.1	19.8 ± 2.6	17.9 ± 3.5	0.07
Type of surgery, n (%)				0.27
-Intra-abdominal surgery	20 (52.6)	9 (42.9)	11 (64.7)	
-Urological surgery	5 (13.2)	2 (9.5)	3 (17.6)	
-Vascular	4 (10.5)	4 (19.0)	0 (0)	
-Limb	4 (10.5)	2 (9.5)	2 (11.8)	
-Spine surgery	2 (5.3)	2 (9.5)	0 (0)	
-Other	3 (7.9)	2 (9.5)	1 (5.9)	
Source of admission, n (%)				
- Operating room	27 (71.1)	17 (81.0)	10 (58.8)	0.14
Emergency surgery, n (%)	7 (18.4)	6 (28.6)	1 (5.9)	0.07
Indication for evaluation, n (%)				
-Hyperlactatemia	25 (65.8)	16 (76.2)	9 (52.9)	0.13
-Hypotension	21 (55.3)	10 (47.6)	11 (64.7)	0.29
-Tachycardia	15 (39.5)	6 (28.6)	9 (52.9)	0.13
-Oliguria	16 (42.1)	6 (28.6)	10 (58.8)	0.06
-Skin mottling	1 (2.6)	1 (4.8)	0 (0)	0.36
Cause of circulatory shock, n (%)				0.11
-Septic shock	21 (55.3)	9 (42.9)	12 (70.6)	
-Hemorrhagic shock	11 (28.9)	9 (42.9)	2 (11.8)	
-Hypovolemic shock	6 (15.8)	3 (14.3)	3 (17.6)	
Vasoactive agents				
-Norepinephrine, n (%)	23 (60.5)	9 (42.9)	14 (82.4)	0.01
-Norepinephrine dose (μg/kg/min)	0.08 (0, 0.18)	0 (0, 0.18)	0.09 (0.08, 0.25)	0.07
- Norepinephrine < 0.1 μg/kg/min, n (%)	25 (65.8)	14 (66.7)	11 (64.7)	0.90
-Epinephrine, n (%)	2 (5.3)	1 (4.8)	1 (5.9)	0.88
-Epinephrine dose (μg/kg/min)	0 (0, 0)	0 (0, 0)	0 (0, 0)	0.93
RASS at baseline	0 (−0.75, 0)	0 (0, 0)	0 (−1, 0)	0.09
Sedative agents				
- Fentanyl, n (%)	31 (81.6)	17 (81.0)	14 (82.4)	0.91
- Fentanyl dose (μg/kg/h)	0.36 (0.22, 0.55)	0.42 (0.22, 0.56)	0.35 (0.23, 0.53)	0.65
Mode of ventilation, n (%)				0.41
- Pressure-controlled ventilation	21 (55.3)	13 (61.9)	8 (47.1)	
- Volume- controlled ventilation	1 (2.6)	0 (0)	1 (5.9)	
- Pressure-support ventilation	15 (39.5)	8 (38.1)	7 (41.2)	
- Other	1 (2.6)	0 (0)	1 (5.9)	
Respiratory parameters				
-pH	7.37 ± 0.07	7.37 ± 0.05	7.38 ± 0.09	0.43
-PaO₂/FiO₂	410.2 ± 144.8	452.6 ± 145.5	357.8 ± 129.4	0.04
-PaCO_2_ (mmHg)	35.3 ± 5.7	35.8 ± 4.2	32.3 ± 4.8	0.08
-PEEP (cmH_2_O)	5 (5, 5)	5 (5, 5)	5 (5, 5)	0.23
-PIP (cmH_2_O)	14 (10.5,16)	14 (10, 16)	16 (12, 16)	0.05
-TV/PBW (mL/kg)	9.2 ± 1.9	8.9 ± 1.8	9.6 ± 1.9	0.22
-MV (L/min)	6.9 (5.9, 8.5)	6.9 (5.7, 8.4)	7.8 (6.0, 8.7)	0.47
Intraabdominal pressure (mmHg)	9.6 ± 1.4	9.5 ± 1.5	9.6 ± 1.4	0.88

Values are presented as mean ± standard deviation (SD), median (25th–75th percentile) or number (percentage)

*BMI* Body mass index, *ASA* American Society of Anesthesiologists, *SOFA* Sequential Organ Failure Assessment, *APACHE II* Acute Physiology and Chronic Health Evaluation II, *HCO₃⁻* bicarbonate, *RASS* Richmond Agitation-Sedation Scale, *PaO₂/FiO₂* arterial oxygen partial pressure to inspired oxygen fraction ratio, *PaCO₂* partial pressure of carbon dioxide, *PEEP* Positive end-expiratory pressure, *PIP* Peak inspiratory pressure, *TV/PBW* Tidal volume per predicted body weight, *MV* Minute ventilation

Non-responders had significantly higher SOFA scores and were more likely to receive norepinephrine. However, the median dose and the proportion of patients receiving a low dose (< 0.1 µg/kg/min) did not differ between groups. No significant differences were observed in APACHE II scores, baseline lactate, or HCO₃⁻ levels. Ventilation mode, sedative use, and IAP were comparable. Among respiratory variables, non-responders had a significantly lower PaO₂/FiO₂ ratio and a trend toward higher PIP, while other parameters (pH, PaCO₂, PEEP, TV/PBW, and MV) did not differ significantly.

### Hemodynamic changes during study interventions

The study protocol and timing of measurements are summarized in Fig. 1. In the overall cohort, T2 compared with T1, was associated with increased MAP and CVP (available for *n =* 27 patients with a central venous catheter), while PPV and SVV decreased. CI remained unchanged, and HR showed a modest reduction. Fluid loading (FL) further increased MAP, CVP, and CI, accompanied by decreases in PPV and SVV. No adverse events occurred during the protocol, and RASS remained stable. Mean ± SD for hemodynamic data is provided in Supplementary Table S1.

**Fig. 3:**
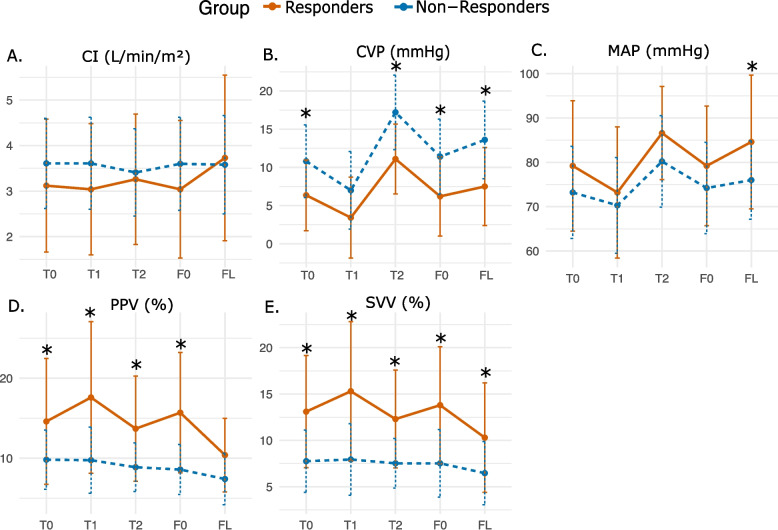
Hemodynamic responses to Trendelenburg maneuver and fluid loading stratified by fluid responsiveness. Changes in (**A**) cardiac index (CI), (**B**) central venous pressure (CVP), (**C**) mean arterial pressure (MAP), (**D**) pulse pressure variation (PPV), and (**E**) stroke volume variation (SVV) across study timepoints (baseline [T0], reverse Trendelenburg [T1], Trendelenburg [T2], pre-fluid loading [F0], and after fluid loading [23]) in fluid responders (solid line) versus non-responders (dashed line). Asterisks (*) indicate a statistically significant difference (*p <* 0.05) between responders and non-responders at that specific time point, determined by post-hoc pairwise comparisons

When stratified by fluid responsiveness, linear mixed-effects models demonstrated distinct hemodynamic trajectories (Fig. 3). Detailed statistical results, including interaction effects and pairwise comparisons, are provided in Supplementary Table S2. A significant group-by-time interaction was observed for CI (*p <* 0.001), reflecting divergent responses to the interventions, although post-hoc analysis did not detect significant between-group differences at specific time points. CVP was significantly higher in non-responders (interaction *p =* 0.006), with confirmed differences at T0, T2, F0, and FL. Similarly, SVV and PPV were consistently higher in responders (interaction *p =* 0.015 and *p =* 0.002, respectively), with significant differences persisting across almost all time points. In contrast, MAP displayed no significant interaction (*p =* 0.094) or overall group effect.

### Comparative hemodynamic responses to the Trendelenburg maneuver

**Fig. 4:**
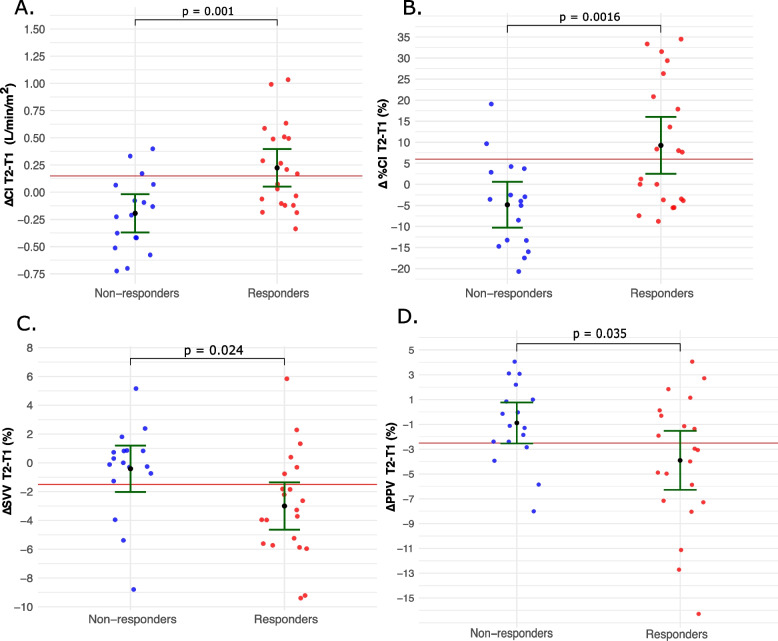
Hemodynamic changes from reverse Trendelenburg (T1) to Trendelenburg (T2) in responders and non-responders. Each panel shows changes in hemodynamic parameters from T2—T1 for individual patients (red dots: responders; blue dots: non-responders). Black dots indicate group means, green bars represent 95% confidence intervals, and the horizontal red line marks the optimal cutoff for predicting fluid responsiveness (see Table 2). *P*-values above each panel reflect between-group differences. **A** absolute change in cardiac index (ΔCI, L/min/m.^2^); (**B**) percent change in cardiac index (Δ%CI, %); (**C**) change in stroke volume variation (ΔSVV, %); (**D**) change in pulse pressure variation (ΔPPV, %)

Figure 4 illustrates hemodynamic changes between the T1 and T2 positions. Responders demonstrated significantly greater increases in both absolute and percent CI (ΔCI and Δ%CI), along with more pronounced reductions in SVV (ΔSVV) and PPV (ΔPPV). CVP increased in both groups, with non-responders showing significantly greater ΔCVP. In contrast, no significant between-group differences were observed for MAP or HR.

As shown in Fig. 4A, there was marked overlap between responders and non-responders across the full ΔCI range, indicating substantial variability in individual responses. Only values above approximately 0.35–0.40 L/min/m^2^ were highly specific for fluid responsiveness, consistent with the broad gray zone observed in Table 2. Detailed numerical data are provided in Appendix II (Supplementary Table S3).

**Table 2:** Diagnostic performance of hemodynamic parameters for predicting fluid responsiveness

Parameter	AUROC (95% CI)	Gray zone | % in gray zone|	Optimal threshold	Sensitivity	Specificity	Positive predictive value	Negative predictive value
ΔCI (T2 − T1), L/min/m^2^	0.78 (0.64–0.93)	− 0.7 to 0.6 | 86.8% **|**	> 0.15ᵃ	52.4% (29.8–74.3%)	82.4% (56.6–96.2%)	78.6% (54.8–91.7%)	58.3% (45.9–69.8%)
			> − 0.35ʸ	100% (100–100%)	41.2% (17.6–64.7%)	67.7% (60.0–77.8%)	100% (100–100%)
Δ%CI (T2 − T1), %	0.76 (0.64–0.91)	− 16.8 to 30.5 | 86.8% **|**	> 6%ᵃ,ʸ	52.4% (29.8–74.3%)	88.2% (63.6–98.6%)	84.6% (58.4–95.6%)	60.0% (48.1–70.8%)
ΔSVV (T2 − T1), %	0.74 (0.57–0.91)	− 5.5 to 1.5 | 86.4% **|**	< − 1.5%ᵃ,ʸ	71.4% (47.8–88.7%)	82.4% (56.6–96.2%)	83.3% (63.4–93.5%)	70.0% (53.4–82.6%)
ΔPPV (T2 − T1), %	0.68 (0.50–0.85)	− 9.5 to 2.5 | 78.9% **|**	< − 2.5%ᵃ,ʸ	57.1% (34.0–78.2%)	76.5% (50.1–93.2%)	75.0% (54.1–88.4%)	59.1% (48.7–80.4%)
Baseline CI (T0), L/min/m^2^	0.70 (0.53–0.87)	2.0 to 5.0 | 81.6% **|**	< 2.85ᵃ,ʸ	57.1% (34.0–78.2%)	82.4% (56.6–96.2%)	80.0% (57.3–92.3%)	60.9% (47.5–72.8%)
Baseline CVP (T0), mmHg	0.77 (0.59–0.95)	2.5 to 15.5 | 81.5% **|**	< 6.5ᵃ,ʸ	71.4% (41.9–91.6%)	76.9% (46.2–95.0%)	76.9% (53.9–90.5%)	71.4% (50.9–85.8%)
Baseline SVV (T0), %	0.76 (0.60–0.91)	4.5 to 20.0 | 84.2% **|**	> 8.5%ᵃ	71.4% (47.8–88.7%)	70.6% (44.0–89.7%)	75.0% (57.8–86.8%)	66.7% (48.8–80.8%)
			> 14.5%ʸ	47.6% (23.8–66.7%)	100% (100–100%)	100% (100–100%)	60.7% (51.5–70.8%)
Baseline PPV (T0), %	0.65 (0.46–0.83)	6.5 to 22.5 | 73.7% **|**	> 11%ᵃ	57.1% (34.0–78.2%)	70.6% (44.0–89.7%)	70.6% (51.3–84.6%)	57.1% (42.7–70.5%)
			> 17.5%ʸ	47.6% (28.6–71.4%)	100% (100–100%)	100% (100–100%)	60.7% (53.1–73.9%)

Values are reported as AUROC (95% confidence interval [95% CI]) or percentage (95% CI), as appropriate. The gray zone indicates the range of values with both sensitivity and specificity below 90%; | % in gray zone | represents the proportion of patients whose results fell within this interval. Sensitivity, specificity, positive predictive value (PPV), and negative predictive value (NPV) are reported at the thresholds shown in the “Optimal threshold” column. Superscript “a” denotes the a priori optimal cut-off selected for the main analysis, whereas superscript “y” denotes the cut-off derived from the Youden index; when both approaches yield the same threshold, this value is labelled with both superscripts

*Abbreviations*: *AUROC* Area under the receiver operating characteristic curve, *T0* baseline in supine position, *T1* baseline in Reverse Trendelenburg position, *T2* after Trendelenburg maneuver, *CI* Cardiac index, *SVV* Stroke volume variation, *PPV* Pulse pressure variation, *CVP* Central venous pressure, *∆CI* absolute change in cardiac index, *∆%CI* percent change in cardiac index, *∆SVV* change in stroke volume variation, *∆PPV* change in pulse pressure variation

### Prediction of fluid responsiveness

**Fig. 5:**
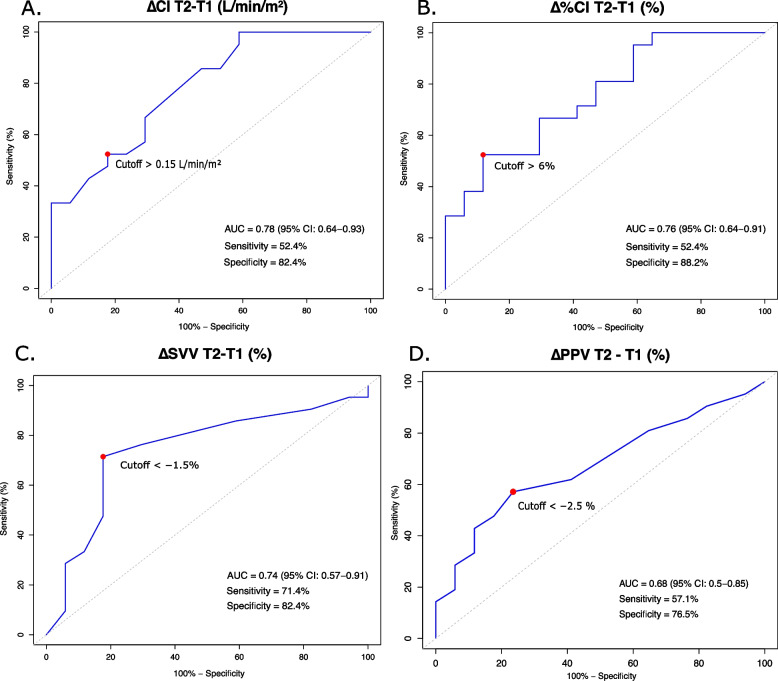
Receiver operating characteristic (ROC) Curves for Trendelenburg-induced hemodynamic changes predicting fluid responsiveness. ROC curves for changes in hemodynamic parameters during the Trendelenburg maneuver to predict fluid responsiveness (*n =* 38). Each panel displays one variable; the red dot marks the optimal cutoff, with corresponding sensitivity, specificity, and area under the curve (AUROC, 95% CI) shown. The diagonal dashed line indicates no discrimination (AUROC = 0.5). **A** Absolute change in cardiac index (ΔCI, L/min/m.^2^); (**B**) percent change in cardiac index (Δ%CI, %); (**C**) change in stroke volume variation (ΔSVV, %); (**D**) change in pulse pressure variation (ΔPPV, %)

The diagnostic performance of Trendelenburg-induced hemodynamic changes (T2–T1) is summarized in Table 2. An absolute increase in CI (ΔCI > 0.15 L/min/m^2^) showed good discrimination for fluid responsiveness, with an AUROC of 0.78 (95% CI 0.64–0.93), sensitivity 52% (29.8–74.3%), and specificity 82% (56.6–96.2%) (Fig. 5A). Similarly, a percent increase in CI (Δ%CI > 6%) achieved an AUROC of 0.76 (95% CI 0.64–0.91), sensitivity 52% (29.8–74.3%), and specificity 88% (63.6–98.6%) (Fig. 5B). A reduction in SVV (ΔSVV) also demonstrated moderate discrimination (AUROC 0.74, 95% CI 0.57–0.91), whereas the change in PPV (ΔPPV) had lower accuracy (AUROC 0.68, 95% CI 0.50–0.85) (Figs. 5C–D).

Gray zone analysis (Table 2 and Supplementary Figure S1) revealed wide diagnostic uncertainty across all variables, with more than 70% of patients within the indeterminate range. For both ΔCI and Δ%CI, over 85% of patients fell within the gray zone, indicating substantial overlap between responders and non-responders despite relatively high AUROC values.

For comparison, ROC curves of baseline parameters (CI, CVP, SVV, and PPV) are provided in Supplementary Figure S2. Among these, baseline CVP and SVV showed the highest discrimination, whereas baseline PPV performed least well.

### Sensitivity analysis

Two sensitivity analyses were performed to assess the robustness of diagnostic performance. First, when fluid responsiveness was defined using a stricter threshold of ≥ 15% increase in CI, AUROC values decreased for ΔCI (0.67; 95% CI 0.49–0.84) and Δ%CI (0.64; 95% CI 0.49–0.82), whereas ΔSVV (0.74; 95% CI 0.56–0.91) and ΔPPV (0.66; 95% CI 0.47–0.85) retained similar discrimination (Supplementary Figure S3).

Second, when the analysis was restricted to patients receiving norepinephrine at < 0.1 µg/kg/min, AUROC values were 0.74 (95% CI 0.55–0.93) for ΔCI, 0.69 (95% CI 0.55–0.90) for Δ%CI, 0.76 (95% CI 0.55–0.96) for ΔSVV, and 0.73 (95% CI 0.52–0.94) for ΔPPV. These results indicate that the overall diagnostic accuracy of Trendelenburg-induced changes was largely preserved under conditions of minimal vasopressor support (Supplementary Figure S4).

## Discussion

Our results showed that, in lightly sedated, mechanically ventilated surgical ICU patients with spontaneous breathing activity, Trendelenburg-induced changes in CI, SVV, and PPV failed to reliably discriminate fluid responsiveness. Even though an absolute increase in CI (ΔCI > 0.15 L/min/m^2^) yielded moderate performance (AUROC 0.78) with high specificity, while a relative increase (Δ%CI > 6%) performed similarly, gray-zone proportions were large. This discrepancy reflects substantial overlap between responders and non-responders across the range of ΔCI values, meaning that AUROC suggests moderate global discrimination but offers little practical value for rule-in or rule-out decisions at the bedside. Sensitivity analyses demonstrated decreased performance when fluid responsiveness was defined as ≥ 15% CI increase, and slightly lower AUROCs when analysis was restricted to patients receiving low-dose norepinephrine, with wide and overlapping confidence intervals across subgroups that reinforce the overall limited and imprecise diagnostic performance of TM.

Previous studies have reported more favorable results for TM-induced hemodynamic changes in different clinical settings. Wang et al. [10] demonstrated that the reverse-Trendelenburg-to-Trendelenburg transition yielded an AUROC of 0.88 (95% CI 0.75–0.96) in fully mechanically ventilated, deeply sedated patients, while Ma et al. [24] reported that velocity time integral (VTI) changes during TM achieved an AUROC of 0.90 (95% CI 0.79–0.96) in the operating room during cardiac surgery. Our ΔCI AUROC of 0.78 (95% CI 0.64–0.93) appears numerically lower, and with the substantially wider gray zone in our cohort (80–90% versus 15–40% in previous studies), this may suggest that the diagnostic value of TM is considerably diminished in patients with spontaneous breathing.

Several physiological and methodological factors likely account for the modest discrimination observed in our cohort. First, our patients were lightly sedated with preserved spontaneous breathing activity (median RASS 0), in whom negative pleural-pressure swings are superimposed on positive-pressure ventilation; this alters preload and afterload and may blunt posture-related venous translocation during TM [28]. Second, the + 10° reverse-Trendelenburg to − 13° Trendelenburg sequence (Δ23°) available on our ICU beds provides a smaller gravitational gradient than the ± 15° configurations (Δ30°) used in prior Trendelenburg studies [10–12]. In Wang et al. [10], a larger angular excursion produced better diagnostic performance, underscoring the importance of tilt magnitude. Physiologically, the volume of venous blood mobilized during postural maneuvers is determined by the hydrostatic pressure gradient, which is proportional to the sine of the tilt angle; smaller angles therefore recruit less unstressed venous volume from the splanchnic and lower-extremity reservoirs [6]. Moreover, compared to PLR which elevates the legs to 45° while keeping the trunk horizontal, mobilizing approximately 300 mL of venous blood from the lower extremities and splanchnic circulation, TM likely mobilizes a smaller volume. Additionally, the cephalad displacement of abdominal contents during TM increases diaphragmatic load and amplifies negative pleural-pressure swings, potentially masking or blunting the hemodynamic response.

In addition to these physiological considerations, our fluid challenge protocol may have limited the ability to detect clear discrimination. We administered 4 mL/kg over 15 min, corresponding to an infusion rate of approximately 1.0–1.3 L/h for a 60–80 kg patient, and assessed the hemodynamic response over a relatively short observation window. Prior work suggests that larger and more rapid fluid challenges, as well as longer post-infusion observation periods, may be required to fully capture a measurable change in cardiac output [21, 29]. An insufficiently intense preload stimulus, combined with a brief monitoring interval, could therefore have attenuated the detectable CI response and contributed to the wide gray zones observed. Finally, CI was derived from an uncalibrated arterial pulse-contour system, which exhibits variable accuracy with changing vascular tone and may introduce additional measurement noise in spontaneously breathing patients. Together, these factors plausibly attenuated the effective preload challenge and contributed to the modest diagnostic discrimination and wider gray zone of TM in this setting.

The impact of vasopressors also requires particular caution when interpreting hemodynamic responses to TM. Monnet et al. [26] reported reduced tracking performance of earlier generation of uncalibrated pulse-contour devices during norepinephrine titration at median doses around 0.16 µg/kg/min. However, an updated fourth-generation algorithm of FloTrac™/Hemosphere™ system has demonstrated improved tracking of cardiac output changes during vasopressor administration, including phenylephrine infusion [30]. Furthermore, prior studies have shown that FloTrac™-guided cardiac output tracking can support effective goal-directed therapy and improve clinical outcomes, supporting its validity for detecting dynamic hemodynamic changes [31, 32]. In our study, although confidence intervals were wide and the findings should be considered exploratory, the sensitivity analyses restricted to patients receiving low-dose norepinephrine (< 0.1 µg/kg/min) showed broadly preserved diagnostic performance. Accordingly, while vasopressor use may complicate the interpretation of preload responsiveness and hemodynamic changes, the limited diagnostic performance observed is more plausibly attributable to physiological and protocol-related factors rather than monitoring technology. Therefore, TM should not be relied upon as a sole indicator of fluid responsiveness, particularly in patients receiving vasopressors, and should be interpreted in conjunction with other dynamic assessments.

When fluid responsiveness was redefined using a stricter cutoff (≥ 15% CI increase), ΔCI performance declined, whereas ΔSVV and ΔPPV remained stable. This divergence likely occurs because SVV and PPV specifically reflect cyclic preload dependency, whereas CI is an integrated measure (affected by preload, afterload, and contractility) that is more susceptible to spontaneous breathing-induced noise [33, 34]. Nevertheless, although specificity remained moderate (> 70%) for both ΔCI > 0.15 L/min/m^2^ and Δ%CI > 6%, these thresholds can at best be considered hypothesis-generating and might have limited utility for identifying fluid responders in highly selected situations. In particular, values of ΔCI above approximately 0.35–0.40 L/min/m^2^ were highly specific for fluid responsiveness, consistent with the upper boundary of the gray zone illustrated in Fig. 4A. However, such findings should be interpreted with caution and not generalized to routine practice without confirmatory evidence from larger studies.

This study has several strengths. To our knowledge, it is the first prospective evaluation of the Trendelenburg maneuver in mechanically ventilated ICU patients with spontaneous breathing activity. A prespecified, standardized protocol with fixed angles and timing was applied, dynamic and baseline indices were assessed concurrently, and a widely available, minimally invasive monitoring platform (FloTrac™/HemoSphere™) was used. Feasibility and safety were demonstrated in a postoperative surgical ICU setting, and gray-zone analysis was included to explicitly characterize diagnostic uncertainty.

Several limitations warrant consideration. First, this was a single-center study with modest sample size and specific surgical exclusions (cardiothoracic, neurosurgical, trauma), which may limit generalizability. Consequently, although the study was powered a priori to detect an AUROC of 0.75, the observed confidence intervals around ΔCI were wide, indicating that the precision of our primary estimate was limited by the sample size. Second, baseline imbalances were present, with non-responders exhibiting higher SOFA scores and more frequent norepinephrine use. Since norepinephrine augments venous return and cardiac output, these patients may have been closer to a hemodynamically optimized state, reducing their likelihood of responding to additional preload. Although sensitivity analyses restricted to low-dose norepinephrine showed similar results, the wide confidence intervals limit the precision of these estimates and residual confounding cannot be excluded. Additionally, the study was not powered to detect differences in secondary outcomes. Third, protocol constraints including bed mechanics limiting tilt to + 10°/− 13° and brief stabilization periods may have attenuated the hemodynamic response. In addition, the absence of quantified measures of spontaneous breathing effort (e.g., P0.1, esophageal pressure swings, diaphragm thickening fraction) limits the mechanistic interpretation of how inspiratory effort influenced the observed hemodynamic changes. Fourth, CI values were displayed as 20-s moving averages sampled over a 1-min interval; this averaging may have smoothed transient peak responses, contributing to the modest AUROC estimates. Finally, the absence of a direct comparison with passive leg raising limits inferences regarding the relative utility of TM in this specific setting.

Future research should validate these findings in larger, multicenter cohorts; standardize key Trendelenburg parameters (including angle, duration, and stabilization time); and incorporate quantitative measures of spontaneous breathing effort to clarify underlying physiological mechanisms. Comparative studies directly evaluating Trendelenburg against passive leg raising are warranted to determine relative diagnostic performance. Future trials should also examine patient-centered outcomes and develop strategies to reduce the wide gray-zone proportion observed in the present study.

## Conclusion

In lightly sedated, mechanically ventilated surgical ICU patients with spontaneous breathing, the Trendelenburg maneuver was feasible and safe but showed limited and inconsistent diagnostic performance for predicting fluid responsiveness. With more than 80% of patients falling within wide indeterminate gray zones, the test is inconclusive for most bedside decisions and should not be used as a stand-alone guide. Given this extent of diagnostic uncertainty, any role for TM in clinical practice appears highly limited and remains uncertain; if used at all, it should be restricted to carefully selected situations and interpreted alongside other dynamic assessments rather than being considered a reliable “supplementary test.” Further head-to-head evaluation against standard methods is warranted.

## Supplementary Information

Additional file 1. This file includes all supplementary materials supporting the main manuscript: Supplementary Appendix I. STARD 2015 checklist. Supplementary Appendix II: Appendix II: Supplementary Table S1. Hemodynamic parameters during the intervention. Appendix II: Supplementary Table S2. Statistical analysis of hemodynamic parameters over time using Linear Mixed-Effects Models. Appendix II: Supplementary Table S3. Comparison of Hemodynamic Changes (T2 - T1) Between Fluid Responders and Non-Responders. Appendix II: Supplementary Figure S1. Gray-zone analysis of diagnostic performance for Trendelenburg-induced hemodynamic changes. Appendix II: Supplementary Figure S2. ROC Curves for Baseline Hemodynamic Parameters (T0) in Predicting Fluid Responsiveness. Appendix II: Supplementary Figure S3. Diagnostic performance of Trendelenburg-induced hemodynamic changes (T2–T1) for predicting fluid responsiveness defined as ≥ 15% increase in cardiac index. Appendix II: Supplementary Figure S4. Diagnostic performance of Trendelenburg-induced hemodynamic changes (T2–T1) in patients receiving low-dose norepinephrine (< 0.1 µg/kg/min).

## Data Availability

The datasets generated and analyzed during the current study are available from the corresponding author on reasonable request.

## Abbreviations

AIC: Akaike Information Criterion
APACHE II: Acute Physiology and Chronic Health Evaluation II
ARDS: Acute Respiratory Distress Syndrome
ASA: American Society of Anesthesiologists
AUROC: Area under the receiver operating characteristic curve
BIC: Bayesian Information Criterion
BMI: Body mass index
CI: Cardiac index
95% CI: 95% Confidence interval
CO₂: Carbon dioxide
CVP: Central venous pressure
Δ (delta): Change (e.g., ΔCI = change in cardiac index)
F0: Pre-infusion measurements.
FL: Post-fluid loading
HCO₃⁻: Bicarbonate
HR: Heart rate
ICU: Intensive care unit
IAP: Intra-abdominal pressure
IQR: Interquartile range
LVEF: Left ventricular ejection fraction
MAP: Mean arterial pressure
MV: Minute ventilation
NPV: Negative predictive value
P0.1: Airway occlusion pressure at 100 ms
PaCO₂: Arterial partial pressure of carbon dioxide
PaO₂/FiO₂: Ratio of arterial oxygen partial pressure to fraction of inspired oxygen
PEEP: Positive end-expiratory pressure
PIP: Peak inspired pressure
PLR: Passive leg raising
PPV: Pulse pressure variation
PPV (diagnostic): Positive predictive value
RASS: Richmond Agitation-Sedation Scale
ROC: Receiver operating characteristic
SpO₂: Peripheral capillary oxygen saturation
SD: Standard deviation
SOFA: Sequential Organ Failure Assessment
SVI: Stroke volume index
SVV: Stroke volume variation
T0: Baseline in the supine position.
T1: Reverse Trendelenburg position
T2: Trendelenburg position
TM: Trendelenburg maneuver
TV/PBW: Tidal volume per predicted body weight
VA-ECMO: Veno-arterial extracorporeal membrane oxygenation
VTI: Velocity time integral
