# Supplementary material for: Trendelenburg maneuver to predict fluid responsiveness in patients under mechanical ventilation with spontaneous breathing: a prospective study
Source: BMC Anesthesiol. 2026 Feb 21;26:196. doi: 10.1186/s12871-026-03706-1 (PMC13032299; doi:10.1186/s12871-026-03706-1)
Supplement: Supplementary file 1 — Additional file 1. This file includes all supplementary materials supporting the main manuscript: Supplementary Appendix I. STARD 2015 checklist. Supplementary Appendix II: Appendix II: Supplementary Table S1. Hemodynamic parameters during the intervention. Appendix II: Supplementary Table S2. Statistical analysis of hemodynamic parameters over time using Linear Mixed-Effects Models. Appendix II: Supplementary Table S3. Comparison of Hemodynamic Changes (T2 - T1) Between Fluid Responders and Non-Responders. Appendix II: Supplementary Figure S1. Gray-zone analysis of diagnostic performance for Trendelenburg-induced hemodynamic changes. Appendix II: Supplementary Figure S2. ROC Curves for Baseline Hemodynamic Parameters (T0) in Predicting Fluid Responsiveness. Appendix II: Supplementary Figure S3. Diagnostic performance of Trendelenburg-induced hemodynamic changes (T2–T1) for predicting fluid responsiveness defined as ≥ 15% increase in cardiac index. Appendix II: Supplementary Figure S4. Diagnostic performance of Trendelenburg-induced hemodynamic changes (T2–T1) in patients receiving low-dose norepinephrine (< 0.1 µg/kg/min). [file 12871_2026_3706_MOESM1_ESM.pdf]

Supplementary Appendix I **Page 2**
STARD 2015 checklist

Supplementary Appendix II:

- Appendix II: Supplementary Table S1 **Page 3**
  Hemodynamic parameters during the intervention
- Appendix II: Supplementary Table S2 **Page 4**
  Statistical analysis of hemodynamic parameters over time using Linear Mixed-Effects Models
- Appendix II: Supplementary Table S3 **Page 4**
  Comparison of Hemodynamic Changes (T2 - T1) Between Fluid Responders and Non-Responders
- Appendix II: Supplementary Figure S1 **Page 5**
  Gray-zone analysis of diagnostic performance for Trendelenburg-induced hemodynamic changes.
- Appendix II: Supplementary Figure S2 **Page 6**
  ROC Curves for Baseline Hemodynamic Parameters (T0) in Predicting Fluid Responsiveness
- Appendix II: Supplementary Figure S3 **Page 7**
  Diagnostic performance of Trendelenburg-induced hemodynamic changes (T2–T1) for predicting fluid responsiveness defined as ≥ 15% increase in cardiac index.
- Appendix II: Supplementary Figure S4 **Page 8**
  Diagnostic performance of Trendelenburg-induced hemodynamic changes (T2–T1) in patients receiving low-dose norepinephrine (< 0.1 μg/kg/min).

Supplementary Appendix I: STARD 2015

the Standards for Reporting of Diagnostic Accuracy Studies (STARD 2015) guidelines

Section & Topic	No	Item	Reported on page #
**TITLE OR ABSTRACT**			
	**1**	Identification as a study of diagnostic accuracy using at least one measure of accuracy (such as sensitivity, specificity, predictive values, or AUC)	2-3
**ABSTRACT**			
	**2**	Structured summary of study design, methods, results, and conclusions (for specific guidance, see STARD for Abstracts)	2-3
**INTRODUCTION**			
	**3**	Scientific and clinical background, including the intended use and clinical role of the index test	4-5
	**4**	Study objectives and hypotheses	5
**METHODS**			
Study design	**5**	Whether data collection was planned before the index test and reference standard were performed (prospective study) or after (retrospective study)	5
Participants	**6**	Eligibility criteria	6
	**7**	On what basis potentially eligible participants were identified (such as symptoms, results from previous tests, inclusion in registry)	6-7
	**8**	Where and when potentially eligible participants were identified (setting, location and dates)	5-6
	**9**	Whether participants formed a consecutive, random or convenience series	5-6
Test methods	**10a**	Index test, in sufficient detail to allow replication	6-8
	**10b**	Reference standard, in sufficient detail to allow replication	8
	**11**	Rationale for choosing the reference standard (if alternatives exist)	8
	**12a**	Definition of and rationale for test positivity cut-offs or result categories of the index test, distinguishing pre-specified from exploratory	9-10
	**12b**	Definition of and rationale for test positivity cut-offs or result categories of the reference standard, distinguishing pre-specified from exploratory	8
	**13a**	Whether clinical information and reference standard results were available to the performers/readers of the index test	6
	**13b**	Whether clinical information and index test results were available to the assessors of the reference standard	6
Analysis	**14**	Methods for estimating or comparing measures of diagnostic accuracy	10-12
	**15**	How indeterminate index test or reference standard results were handled	10-11
	**16**	How missing data on the index test and reference standard were handled	-
	**17**	Any analyses of variability in diagnostic accuracy, distinguishing pre-specified from exploratory	10-12
	**18**	Intended sample size and how it was determined	10
**RESULTS**			
Participants	**19**	Flow of participants, using a diagram	12, figure 2
	**20**	Baseline demographic and clinical characteristics of participants	12, table 1
	**21a**	Distribution of severity of disease in those with the target condition	12, table 1
	**21b**	Distribution of alternative diagnoses in those without the target condition	12
	**22**	Time interval and any clinical interventions between index test and reference standard	Figure 1
Test results	**23**	Cross tabulation of the index test results (or their distribution) by the results of the reference standard	12-13, table 2
	**24**	Estimates of diagnostic accuracy and their precision (such as 95% confidence intervals)	13-14
	**25**	Any adverse events from performing the index test or the reference standard	13
**DISCUSSION**			
	**26**	Study limitations, including sources of potential bias, statistical uncertainty, and generalisability	18-19
	**27**	Implications for practice, including the intended use and clinical role of the index test	15-20
**OTHER INFORMATION**			
	**28**	Registration number and name of registry	23
	**29**	Where the full study protocol can be accessed	-
	**30**	Sources of funding and other support; role of funders	23

Supplementary Appendix II: Supplementary Table S1. Hemodynamic parameters during the intervention.

Parameter	Baseline-1 (T0)	Reverse Trendelenburg 10° (T1)	Trendelenburg -13° (T2)	p-value between T1–T2	Baseline-2: Before fluid loading (F0)	After fluid loading (FL)	p-value between F0–FL
**MAP (mmHg)**	76.5±13.1	71.9±13.1	83.7±10.7	< 0.001*	77.0±12.3	80.7±13.3	< 0.001*
- Responders	79.2±14.7	73.2±14.8	86.6±10.5	< 0.001*	79.2±13.5	84.6±15.1	< 0.001*
- Non-Responders	73.2±10.4	70.3±10.8	80.2±10.3	< 0.001*	74.2±10.3	76.0±8.9	0.24
**Heart rate (bpm)**	96.4±17.2	96.8±17.2	95.1±16.8	0.007*	95.1±17.5	93.9±15.8	0.28
- Responders	93.1±17.4	93.7±16.8	92.0±16.3	0.12	91.2±17.6	91.6±14.2	0.78
- Non-Responders	100.0±16.7	101.0±17.4	98.8±17.2	0.009*	99.9±16.7	96.8±17.6	0.03
**CVP (mmHg) (n = 27)**	8.5±5.1	5.2±5.4	14.0±5.6	< 0.001*	8.7±5.6	10.4±5.9	< 0.001*
- Responders (n= 14)	6.4±4.6	3.4±5.3	11.1±4.6	< 0.001*	6.2±5.2	7.5±5.1	< 0.001*
- Non-Responders (n= 13)	10.8±4.8	7.0±5.1	17.2±4.9	< 0.001*	11.4±4.9	13.6±5.1	< 0.001*
**PPV (%)**	12.5±6.7	14.1±8.5	11.5 ±5.8	0.002*	12.5±6.9	9.1±4.3	< 0.001*
- Responders	14.6±7.9	17.6±9.5	13.7±6.6	0.002*	15.7±7.5	10.4±4.6	< 0.001*
- Non-Responders	9.8±3.7	9.8±4.1	8.9±3.0	0.28	8.6±3.1	7.4±3.2	0.09
**SVV (%)**	10.7±5.6	12.0±7.1	10.2±4.9	0.003*	11.0±6.1	8.6±5.3	< 0.001*
- Responders	13.1±6.1	15.3±7.5	12.3±5.3	0.001*	13.8±6.3	10.3±5.9	0.004*
- Non-Responders	7.8±3.3	7.9±3.9	7.5±2.7	0.60	7.5±3.6	6.5±3.4	0.05
**CI (L/min/m²)**	3.34±1.28	3.29±1.28	3.33±1.23	0.58	3.29±1.32	3.66±1.52	< 0.001*
- Responders	3.12±1.46	3.04±1.44	3.26±1.43	0.01*	3.04±1.51	3.73±1.82	< 0.001*
- Non-Responders	3.61±0.99	3.61±1.01	3.41±0.96	0.03*	3.60±1.02	3.58±1.08	0.63

Values are reported as the mean ± standard deviation (SD). *$p < 0.05$ was considered statistically significant. MAP: mean arterial pressure; CVP: central venous pressure; PPV: pulse pressure variation; SVV: stroke volume variation; CI: cardiac index.

Supplementary Table S2 : Statistical analysis of hemodynamic parameters over time using Linear Mixed-Effects Models

Parameter	Interaction (Group × Time) P-value	T0 (Baseline)	T1 (Reverse Trend)	T2 (Trendelenburg)	F0 (Pre-fluid)	FL (Post-fluid)
CI	< 0.001	0.268	0.199	0.732	0.207	0.728
CVP	0.006	0.028	0.071	0.003	0.011	0.003
MAP	0.094	0.145	0.475	0.119	0.220	0.038
SVV	0.015	0.003	< 0.001	0.007	< 0.001	0.028
PPV	0.002	0.017	< 0.001	0.017	< 0.001	0.133

Values displayed are P-values derived from linear mixed-effects models. Bold values indicate statistical significance ($p < 0.05$). Abbreviations: CI: cardiac index; CVP: central venous pressure; MAP: mean arterial pressure; PPV: pulse pressure variation; SVV: stroke volume variation; T0: baseline (supine); T1: reverse Trendelenburg; T2: Trendelenburg; F0: pre-fluid loading (supine); FL: post-fluid loading.

Supplementary Table S3. Comparison of hemodynamic changes (T2 – T1) between fluid responders and non-responders.

Parameter	Responder group	Non-responder group	p-value
ΔCI T2 - T1 (L/min/m²)	0.22 ± 0.38	-0.19 ± 0.34	<0.001*
Δ%CI T2 - T1 (%)	9.26 ± 14.87	-4.85 ± 10.60	0.0016*
ΔSVV T2 - T1 (%)	-3.0 ± 3.6	-0.4 ± 3.1	0.02*
ΔPPV T2 - T1 (%)	-3.9 ± 5.2	-0.9 ± 3.2	0.04*
ΔCVP T2 - T1 (mmHg)	7.6 ± 3.4	10.2 ± 2.6	0.04*
ΔMAP T2 - T1 (mmHg)	13.4 ± 7.4	9.9 ± 5.6	0.110
ΔHR T2 - T1 (bpm)	-1.7 ± 4.8	-1.9 ± 2.7	0.82

Values are reported as the mean ± standard deviation (SD). *$P < 0.05$ indicates statistical significance. T1: reverse Trendelenburg position; T2: after Trendelenburg positioning. ΔCI: change in cardiac index (T2–T1); Δ%CI: percent change in cardiac index from T2 - T1; ΔSVV: change in stroke volume variation (T2–T1); ΔPPV: change in pulse pressure variation (T2–T1); CVP: central venous pressure; ΔCVP: change in central venous pressure (T2–T1); ΔMAP: change in mean arterial pressure (T2–T1); ΔHR: change in heart rate (T2–T1).

Supplementary Figure S1. Gray-zone analysis of diagnostic performance for Trendelenburg-induced hemodynamic changes.

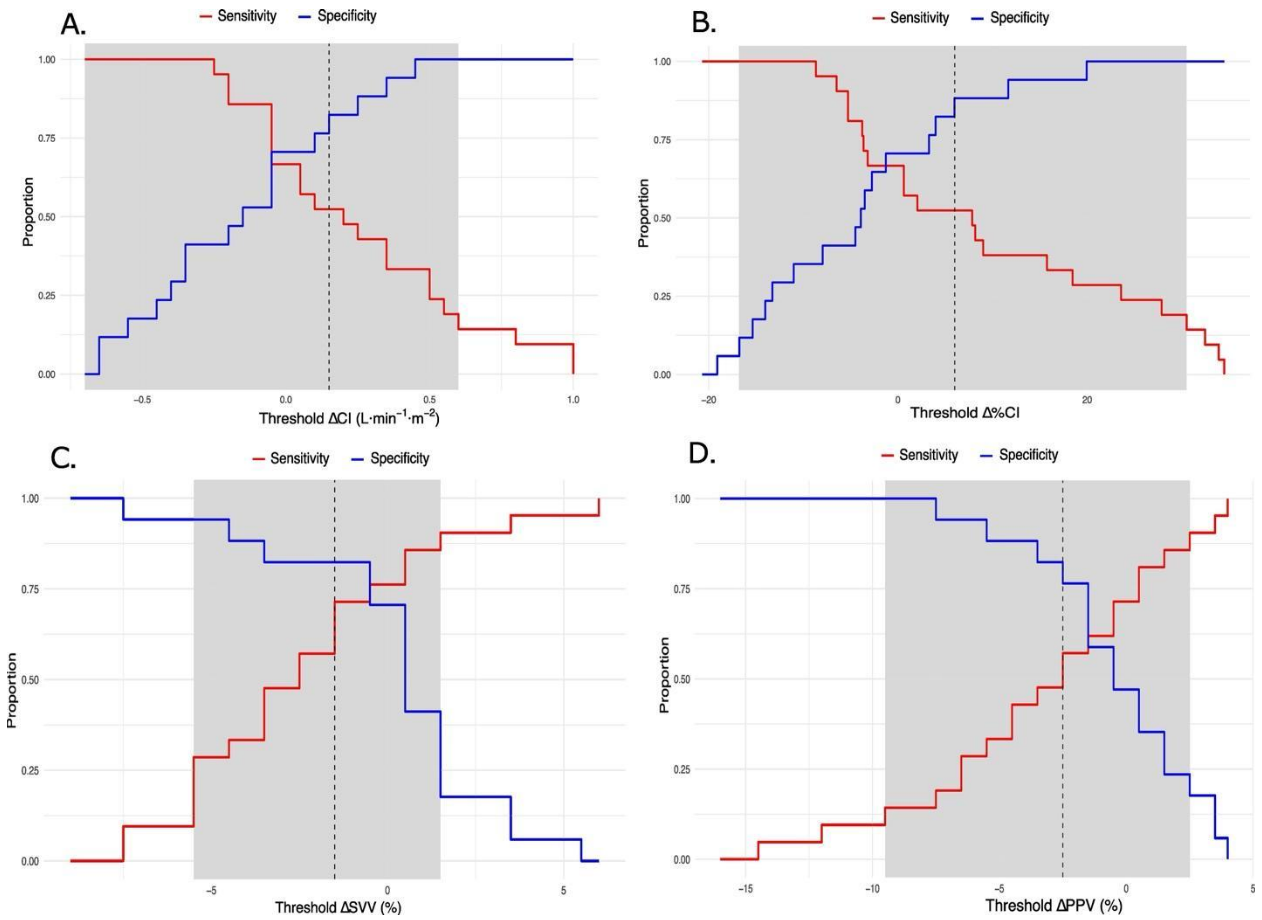

Each panel displays the relationship between sensitivity (red line) and specificity (blue line) across thresholds of the tested parameter, with the shaded gray area indicating the gray zone and the dashed line marking the optimal cutoff value.

(A) ΔCI (T_2–T_1): absolute change in cardiac index (L/min/m²).

(B) Δ%CI (T_2–T_1): percent change in cardiac index (%).

(C) ΔSVV (T_2–T_1): change in stroke volume variation (%).

(D) ΔPPV (T_2–T_1): change in pulse pressure variation (%).

CI = cardiac index; SVV = stroke volume variation; PPV = pulse pressure variation.

Supplementary Figure S2. The receiver operating characteristic (ROC) curves for baseline hemodynamic parameters (T0) in predicting fluid responsiveness in mechanically ventilated patients with spontaneous breathing activity.

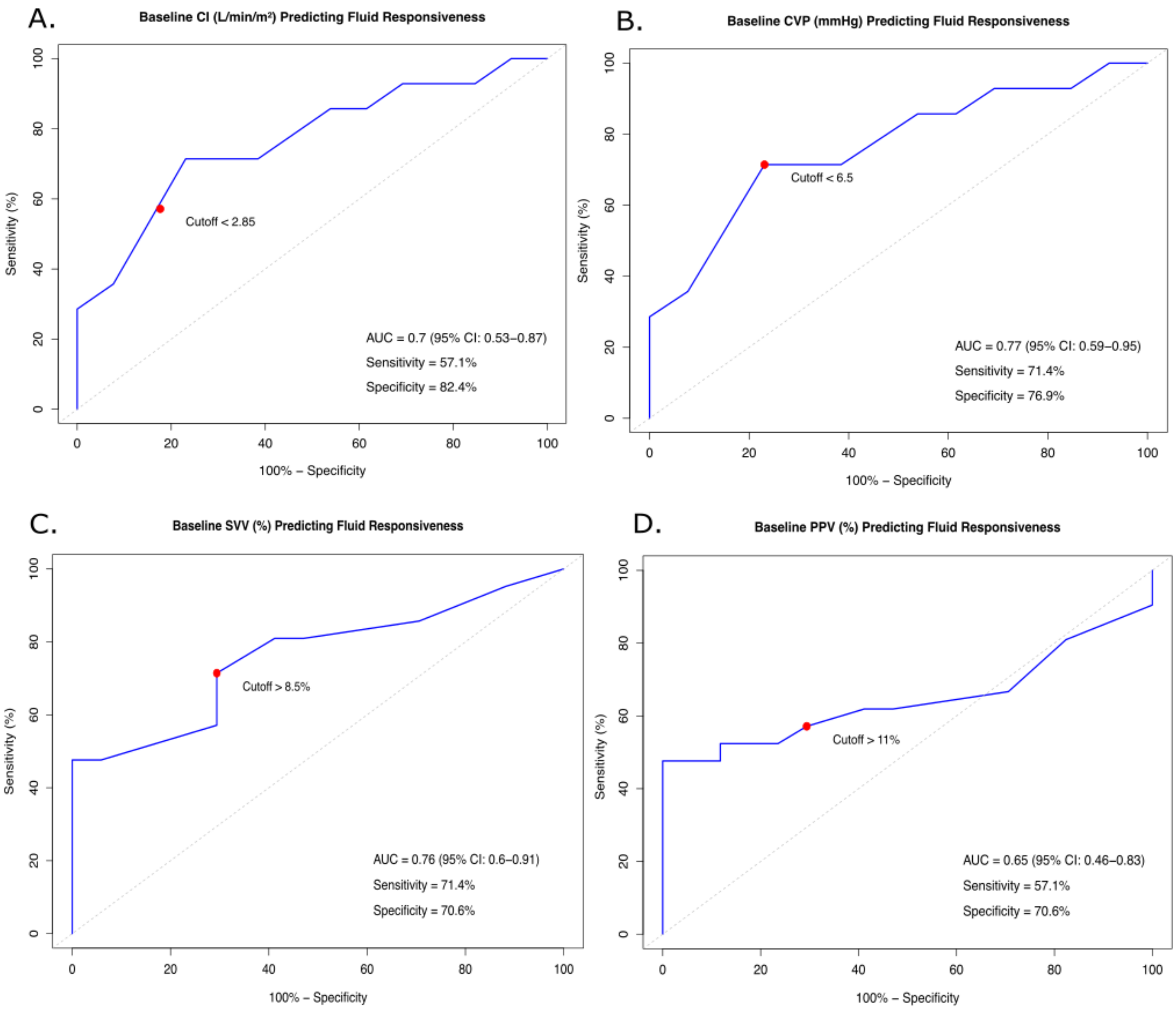

ROC curves for baseline (T0) hemodynamic parameters in predicting fluid responsiveness (n = 38). (A) Cardiac index (CI, L/min/m²). (B) Central venous pressure (CVP, mmHg). (C) Stroke volume variation (SVV, %). (D) Pulse pressure variation (PPV, %). The red dot indicates the optimal cutoff value for each variable. The area under the curve (AUC) with 95% confidence intervals, along with sensitivity and specificity, are displayed within each panel. The dashed diagonal line represents the reference (AUC = 0.5).

Supplementary Figure S3. Diagnostic performance of Trendelenburg-induced hemodynamic changes (T2–T1) for predicting fluid responsiveness defined as ≥ 15% increase in cardiac index.

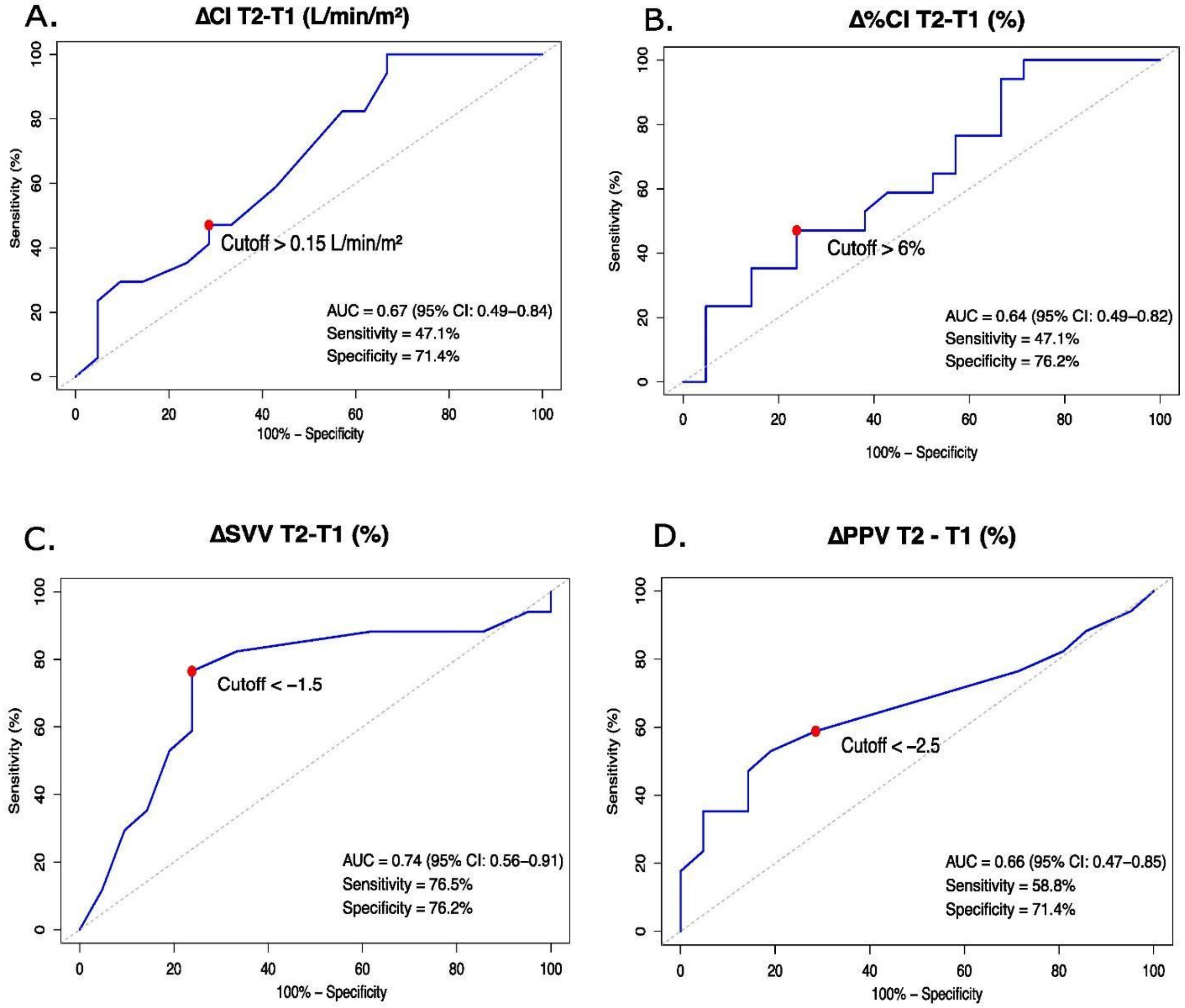

Receiver-operating characteristic (ROC) curves are shown for (A) ΔCI, (B) Δ%CI, (C) ΔSVV, and (D) ΔPPV. AUROC values with 95% confidence intervals are presented in each panel. CI = cardiac index (L/min/m²); SVV = stroke volume variation (%); PPV = pulse pressure variation (%).

Supplementary Figure S4. Diagnostic performance of Trendelenburg-induced hemodynamic changes (T2–T1) in patients receiving low-dose norepinephrine (< 0.1 μg/kg/min).

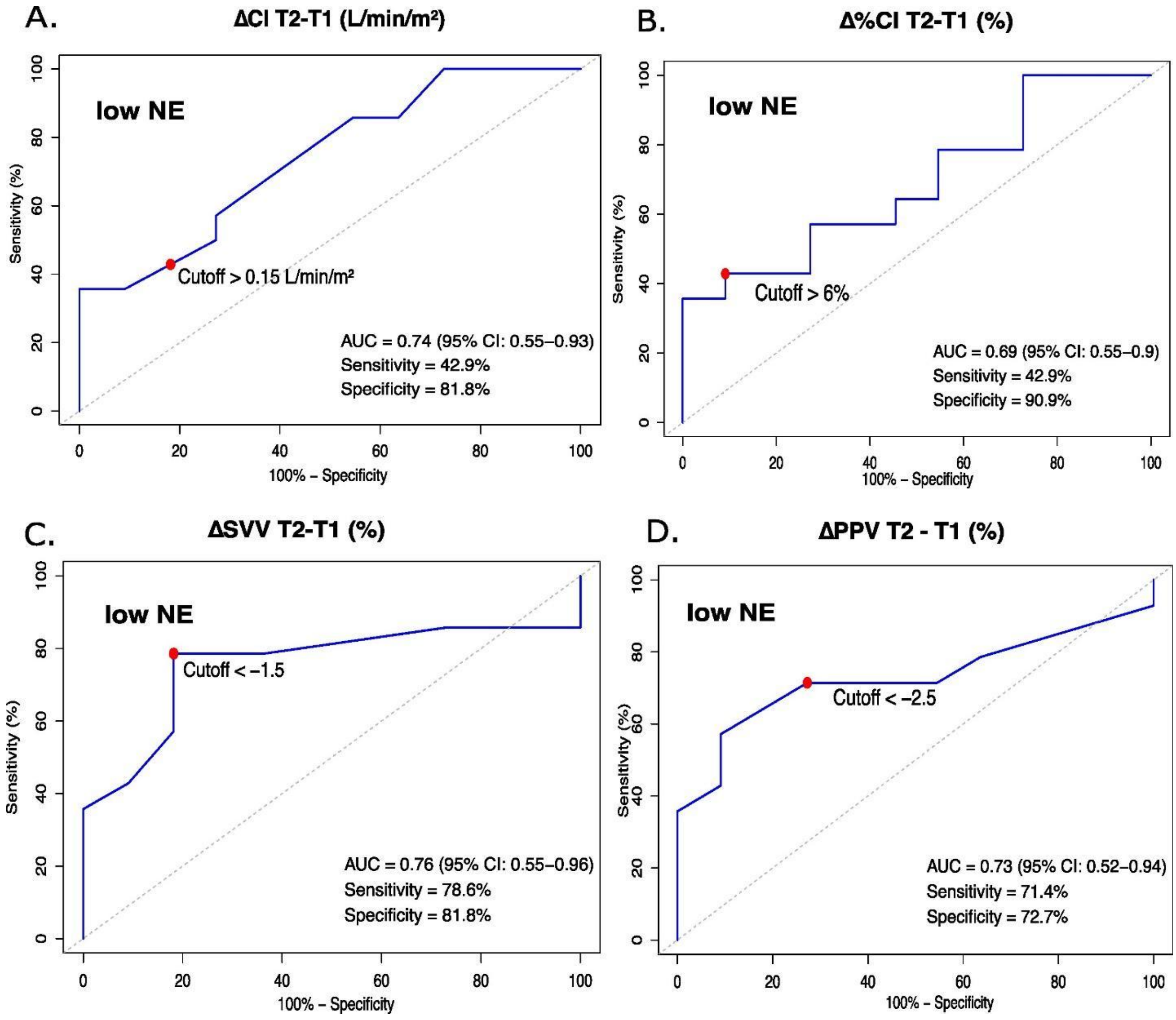

Receiver-operating characteristic (ROC) curves are shown for (A) ΔCI, (B) Δ%CI, (C) ΔSVV, and (D) ΔPPV. AUROC values with 95% confidence intervals are presented in each panel. CI = cardiac index (L/min/m²); SVV = stroke volume variation (%); PPV = pulse pressure variation (%).
